# Estrogen-Mediated Regulation of Fam3d in Mouse Uterus During the Estrous Cycle

**DOI:** 10.3390/ijms262411840

**Published:** 2025-12-08

**Authors:** Hyukjung Kim, Byeongseok Kim, Joohee Kim, Yeonju Suh, Jimin Lee, Sangok Park, Man Ryul Lee, Hoi Chang Lee, Youngsok Choi

**Affiliations:** 1 Department of Stem Cell and Regenerative Biotechnology, Konkuk University, Seoul 05029, Republic of Korea; mykhj102@naver.com (H.K.); qufsksrlaqud@naver.com (B.K.); ysjh06029@naver.com (J.K.); syj93129@naver.com (Y.S.); 3136jm@naver.com (J.L.); sangok731@naver.com (S.P.); 2 Major in Stem Cell and Regenerative Biotechnology, School of Advanced Biotechnology, Konkuk University, Seoul 05029, Republic of Korea; stemopia@konkuk.ac.kr; 3 The Institute of Advanced Regenerative Sciences, Konkuk University, Seoul 05029, Republic of Korea; 4 Department of Obstetrics and Gynecology, Feinberg School of Medicine, Northwestern University, Chicago, IL 60611, USA; hoi.lee@northwestern.edu

**Keywords:** uterus, estrous cycle, estrogen, family with sequence similarity 3

## Abstract

In mice, the uterus undergoes dynamic changes regulated by estrogen and progesterone during the estrous cycle. Proper regulation of these changes is critical for successful pregnancy. The Family with sequence similarity 3 (Fam3) gene family, comprising *Fam3a*, *Fam3b*, *Fam3c*, and *Fam3d*, encodes cytokine-like proteins, but their uterine roles remain unclear. This study examined Fam3 expression in the mouse uterus across the estrous cycle and assessed estrogen-dependent regulation. RNA-seq analysis revealed increased *Fam3b*, *Fam3c*, and *Fam3d* expression during proestrus and estrus. Notably, *Fam3d* showed dynamic regulation, peaking in these stages. To test estrogen regulation, estradiol was administered to ovariectomized mice, showing maximal *Fam3d* expression at 24 h post-injection. ERα antagonist treatment blocked this induction, indicating ERα-mediated regulation. Immunofluorescence localized FAM3D to the cytoplasm of luminal and glandular epithelia, especially in the apical region, with no stromal or nuclear expression. These findings suggest that estrogen and Erα (Estrogen receptor alpha) signaling control Fam3d expression, implicating FAM3D in uterine epithelial function. This study provides novel insights into *Fam3d*’s role in uterine physiology and a foundation for exploring its function in reproduction.

## 1. Introduction

The female reproductive organ, the uterus, undergoes cyclic changes that require morphological and histological regulation of the endometrium. These changes and their accurate regulation are crucial for a successful pregnancy [1]. The human menstrual cycle occurs approximately every 28–30 days, whereas its counterpart in rodents, the estrous cycle, lasts for 4–5 days and comprises four stages: proestrus, estrus, metestrus, and diestrus [2,3]. Proestrus, the initial stage of the reproductive cycle, is characterized by follicular growth in the ovary, preparing the ovulation. Estrus, also known as the “heat” period, is the phase during which the animal is sexually receptive and can conceive. Metestrus, a relatively short period following estrus, is characterized by a gradual decrease in reproductive organ activity if conception does not occur. Diestrus refers to a functional period of sexual inactivity and uterine remodeling before the next reproductive cycle [4]. The transition from proestrus to estrus is marked by a significant increase in estrogen levels. In contrast, the diestrus phase is characterized by elevated progesterone but basal estrogen levels [5].

Estrogen, a hormone belonging to the class of female steroids, is essential for the development and regulation of the female reproductive system [6]. It is primarily produced in the ovaries and exerts specific effects on target tissues via genomic or non-genomic mechanisms [7]. The actions of estrogen are mediated by nuclear estrogen receptors (ERs) present in target organs. ER proteins are classified into estrogen receptor alpha (ERα) and estrogen receptor beta (ERβ), and their expression patterns vary across tissues and cell types. In the uterus, ERα is predominantly expressed and influences the response to estrogen [8]. It is present in uterine cells and promotes their growth and differentiation.

The gene family with sequence similarity 3 member D (FAM3D) encodes a protein belonging to a cytokine-like family characterized by a four-helix bundle structure. The other members of this family are FAM3A, FAM3B, and FAM3C [9]. FAM3D is predominantly expressed in the gastrointestinal tract [10]. It plays a vital role in homeostasis, protection against inflammation-associated cancer, and the maintenance of normal microbiota composition in the colon [11]. It has been identified as a host-derived chemotactic agonist of formyl peptide receptors (FPR1 and FPR2), suggesting potential pro-inflammatory properties [12,13]. However, the expression and regulation of FAM3D in other tissues, including the uterus, remain unknown. Moreover, regulation of the Fam3 family members in mouse uterine physiology remains largely unknown. We hypothesized that the expression of Fam3 family members, particularly *Fam3d*, is dynamically regulated during the estrous cycle in a manner dependent on estrogen signaling. Therefore, we investigated the expression of FAM3D in the uterus during the estrous cycle and evaluated the regulation of its expression by the ovarian steroid hormone, estrogen, using a mouse model.

## 2. Results

### 2.1. Gene Profile of Mouse Uterus During the Estrous Cycle

**Figure 1:**
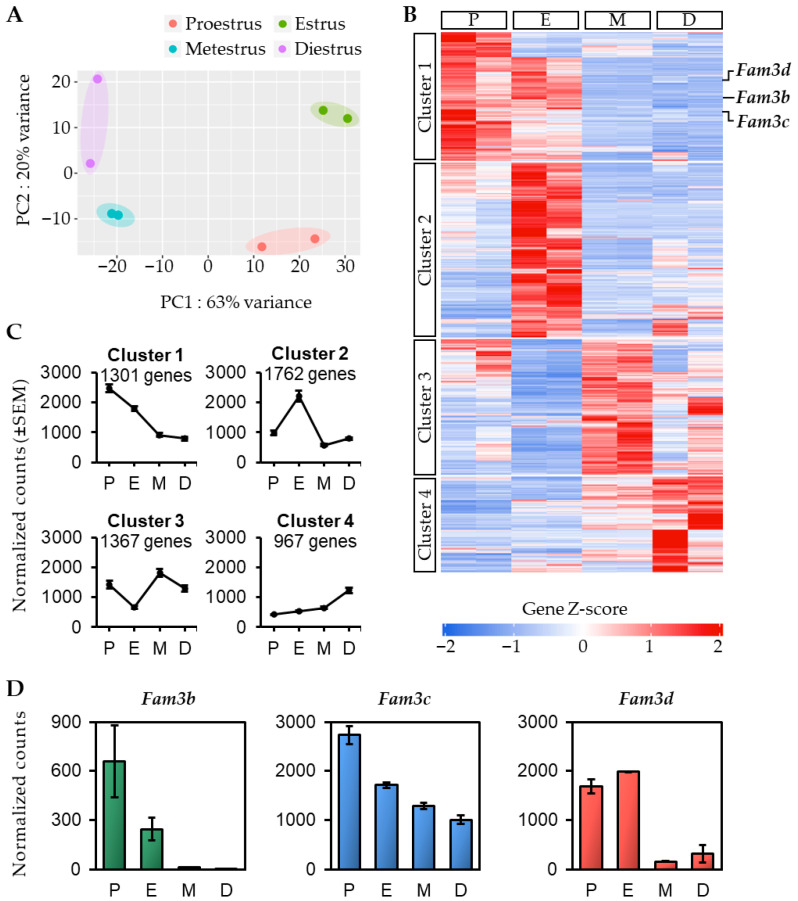
Transcriptional profile indicating differentially expressed genes in mouse uterus during estrous cycle. (**A**) Principal component analysis (PCA) plot of RNA-seq samples showing distinct separation of each group. (**B**) Heatmap indicating differentially expressed genes during estrous cycle. Each cluster was classified based on dynamic changes in its expression. Red color indicates a relative increase in gene expression, blue color indicates decreased expression. (**C**) The expression patterns of each cluster during estrous cycle. Standard error of the mean (SEM) was calculated for each group. (**D**) The expression levels of *Fam3b*, *Fam3c*, and *Fam3d* during estrous cycle based on RNA-seq data. P, proestrus; E, estrus; M, metestrus; D, diestrus.

The proestrus, estrus, metestrus, and diestrus stages of the estrous cycle were determined using vaginal smear assay (Supplementary Figure S1). The differentially expressed genes in the mouse uterus during the different stages of the estrous cycle were identified by RNA-seq analysis. PCA using normalized read counts effectively demonstrated variability among groups and showed that the samples within each group clustered together (Figure 1A). This indicates distinct transcriptomic differences between the stages of the estrous cycle.

Next, we performed heat map analysis for the genes showing significant differences in expression between each stage. Using the criteria of fold change >2 and FDR <0.05, we obtained 5397 genes, which were classified into four different clusters according to their expression patterns during the estrous cycle (Figure 1B,C). Gene Ontology (GO) analysis was performed to determine the biological processes in which the genes in each cluster were enriched (Supplementary Figure S2). Cluster1 (1301 genes) exhibited strong expression during the proestrus stage, which gradually decreased as the estrous cycle progressed. The major regulated biological processes were related to the immune system and lipid metabolism. Cluster2 (1762 genes) showed high expression during the estrus stage, in contrast to low expression in other stages. It was also associated with the immune system and inflammatory responses. Cluster3 (1367 genes) displayed a significant decrease in expression from proestrus to estrus, followed by a notable increase during metestrus. It was associated with cell cycle and cell division. Cluster4 (967 genes) showed minimal expression during the proestrus stage; however, the expression gradually increased as estrous cycle progressed and was relatively higher during diestrus. It was associated with the organization of the extracellular matrix and development of multicellular organisms.

Evaluation of the Fam3 gene family, comprising *Fam3a*, *Fam3b*, *Fam3c*, and *Fam3d*, revealed that *Fam3a* expression did not significantly vary throughout the estrous cycle, whereas the remaining members were classified into Cluster 1. Quantification using normalized counts revealed that *Fam3b*, *Fam3c*, and *Fam3d* were highly expressed during the proestrus stage (Figure 1D). Therefore, we focused on *Fam3b*, *Fam3c*, and *Fam3d* in the subsequent experiments.

### 2.2. Expression of FAM3 Gene Family in Mouse Uterus During the Estrous Cycle

**Figure 2:**
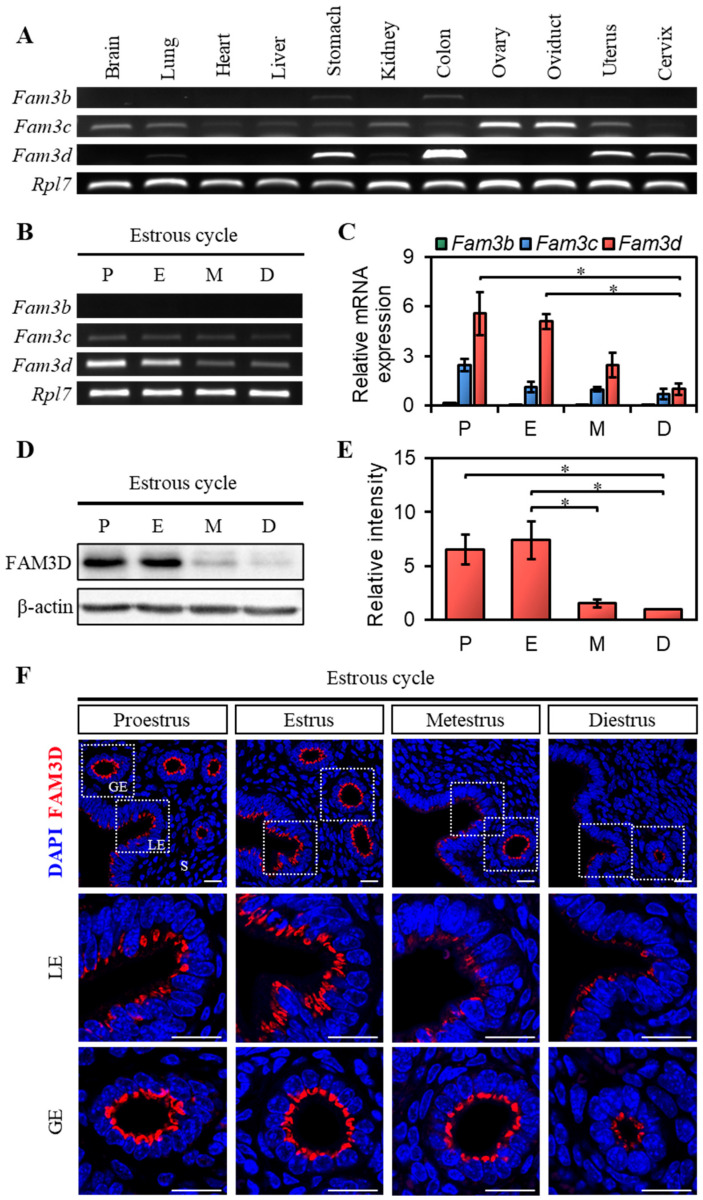
Expression of Fam3 family in different mouse organs and the uterus during estrous cycle. (**A**) RT-PCR analysis of Fam3 family in different mouse tissues. Ribosomal protein L7 (*Rpl7*) was used as an internal control. (**B**) RT-PCR analysis of Fam3 family in mouse uterus during each stage of the estrous cycle. *Rpl7* was used as an internal control. (**C**) qRT-PCR analysis of Fam3 family in mice uteri for each stage of the estrous cycle. Standard error of the mean (SEM) was calculated for each group (*n* = 3). The fold changes were evaluated by comparing the mRNA expression level at each stage to *Fam3d* expression at diestrus. The statistical significance was determined by one-way ANOVA followed by Tukey’s multiple comparison test (* *p* < 0.05). (**D**) Western blot analysis of FAM3D in mouse uterus during each stage of the estrous cycle. β-actin was used as an internal control. (**E**) Relative intensity of FAM3D in mouse uterus during each stage of the estrous cycle. Relative intensities were normalized to that of β-actin. SEM was calculated for each group (*n* = 3). The fold changes were evaluated by comparing the expression level at each stage to that at diestrus. The statistical significance was determined by one-way ANOVA followed by Tukey’s multiple comparison test (* *p* < 0.05). (**F**) Immunofluorescence staining of mouse uterus at each stage during the estrous cycle using FAM3D antibody (*n* = 3). Red represents FAM3D and blue represents DAPI. Scale bar, 20 μm. LE, luminal epithelium; GE, glandular epithelium; S, stroma; P, proestrus; E, estrus; M, metestrus; D, diestrus.

The expression patterns of the Fam3 gene family were investigated in detail by RT-PCR, targeting *Fam3b*, *Fam3c*, and *Fam3d* in the murine female reproductive system and other organs (Figure 2A). *Fam3b* expression was detected in the stomach and colon, but its expression was relatively low compared to that of the other two genes. *Fam3c* expression was higher in the ovaries and oviducts than in other organs. *Fam3d* expression was the highest among all three genes in the uterus, cervix, colon, and stomach. Next, we examined *Fam3b*, *Fam3c*, and *Fam3d* expression in the mouse uterus during the estrous cycle (Figure 2B,C). *Fam3b* and *Fam3c* showed relatively low expression, whereas *Fam3d* exhibited robust expression compared to the other members. The transcription level of *Fam3d* was high during the proestrus and estrus stages but decreased during the metestrus and diestrus stages. Therefore, we further analyzed the expression pattern of FAM3D in the uterine endometrium.

Western blot analysis confirmed that FAM3D expression in the mouse uterus is dynamically regulated during the estrous cycle, similar to its RNA expression pattern. FAM3D showed high expression during the proestrus and estrus stages and significantly lower expression during the metestrus and diestrus stages (Figure 2D,E). Furthermore, we examined the localization of FAM3D in the uterus by immunofluorescence staining. FAM3D was exclusively expressed in the luminal epithelium (LE) and glandular epithelium (GE) but not in the stroma (Figure 2F). Notably, FAM3D was observed only in the cytoplasm, mainly concentrated in the apical region, but not in the nucleus. Its expression varied between the different stages of the estrous cycle. FAM3D showed high expression in LE during the proestrus and estrus stages and lower expression during the metestrus and diestrus stages. In contrast, GE showed high expression of FAM3D during the proestrus and estrus stages and remained relatively strong through metestrus before declining at diestrus. These results indicate that both the mRNA and protein expression of *Fam3d* were dynamically regulated in the mouse uterus during the estrous cycle, with high expression during the proestrus and estrous stages.

### 2.3. Expression of FAM3D in E2-Treated OVX Mouse Uterus

**Figure 3:**
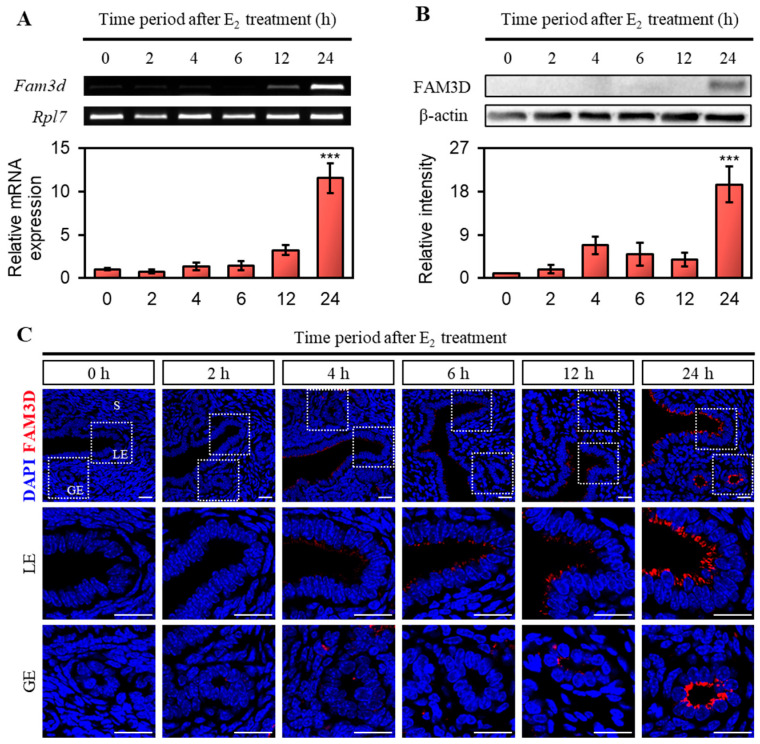
Expression of *Fam3d* in the uterus of ovariectomized (OVX) mice following treatment with E2. (**A**) RT-PCR and qRT-PCR analysis of *Fam3d* in OVX mouse uterus at 2, 4, 6, 12, and 24 h post-E2 treatment. Ribosomal protein L7 (*Rpl7*) was used as an internal control. Standard error of the mean (SEM) was calculated for each group (*n* = 3). The fold changes were determined by comparing the mRNA expression levels at each time point to the levels of 0 h samples. The statistical significance was determined by one-way ANOVA followed by Tukey’s multiple comparison test (*** *p* < 0.001 versus 0 h). (**B**) Western blot analysis of FAM3D in OVX mouse uterus at 2, 4, 6, 12, and 24 h post-E2 treatment. β-actin was used as an internal control. Relative intensities were normalized to that of β-actin. SEM was calculated for each group (*n* = 3). The fold changes were evaluated by comparing the expression levels at each time point to the levels of 0 h samples. The statistical significance was determined by one-way ANOVA followed by Tukey’s multiple comparison test (*** *p* < 0.001 versus 0 h). (**C**) Immunofluorescence staining of the OVX mice uteri at 2, 4, 6, 12, and 24 h after E2 treatment using FAM3D antibody (*n* = 3). Red represents FAM3D and blue represents DAPI. Scale bar, 20 μm. LE, luminal epithelium; GE, glandular epithelium; S, stroma.

As estrogen levels were significantly elevated during the proestrus and estrus stages of the estrous cycle, we investigated whether *Fam3d* expression is affected by estrogen. To exclude the influence of endogenous ovarian hormones, we used OVX mice and evaluated changes in *Fam3d* expression after E2 treatment over time. Initially, *Fam3d* mRNA was not induced by E2; however, we observed a sharp increase in *Fam3d* transcription in the uteri of OVX mice 24 h after E2 treatment (Figure 3A).

Next, we analyzed the protein expression and localization of FAM3D in OVX mouse uterus after E2 treatment using Western blotting and immunofluorescence. Consistent with the mRNA expression, FAM3D protein expression in the uterus of OVX mice significantly increased after 24 h of E2 treatment (Figure 3B). Furthermore, similar to the patterns observed in the normal uterus, FAM3D was exclusively expressed in the apical region of the LE and GE of the uterine endometrium, with robust expression 24 h after E2 treatment (Figure 3C). These results indicated that mRNA and protein expression of *Fam3d* were regulated by estrogen.

### 2.4. Estrogen-Dependent Expression of Fam3d Is Mediated via ER

**Figure 4:**
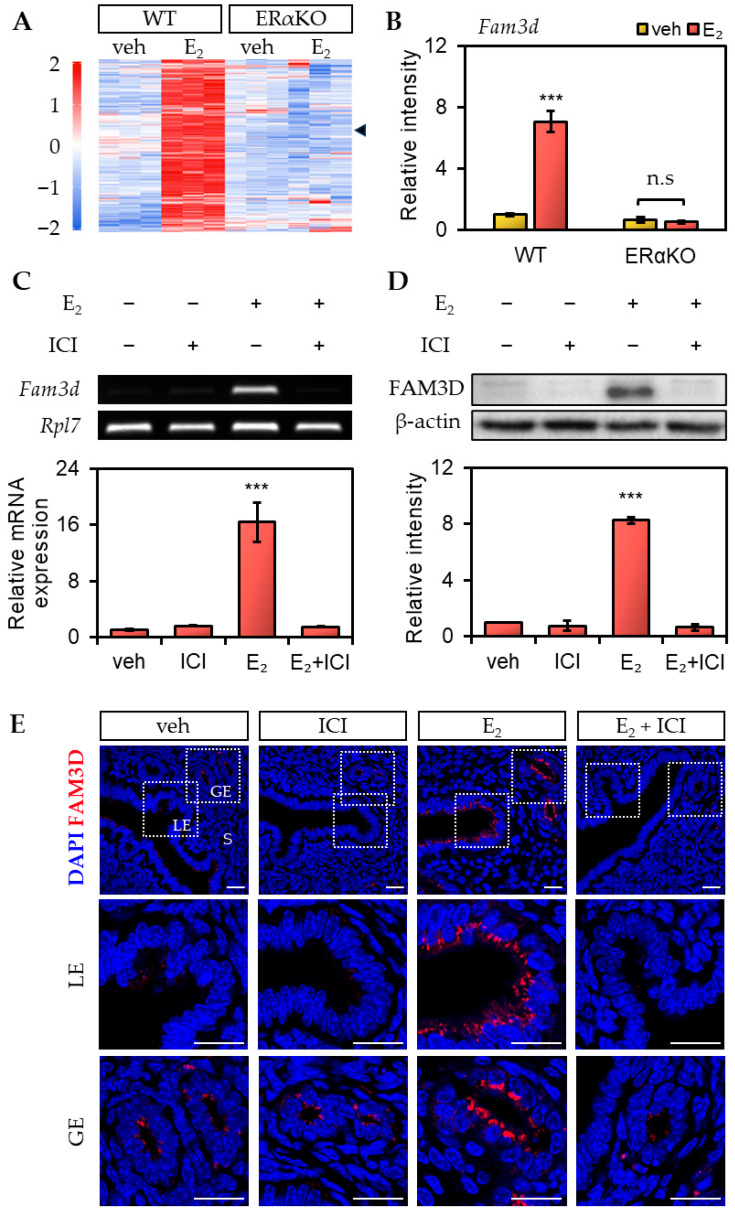
Expression of *Fam3d* in the uterus of ovariectomized (OVX) mice at 24 h after treatment with estrogen receptor alpha (ERα) antagonist, ICI. (**A**,**B**) Heatmap indicating differentially expressed genes in ERα knockout OVX mouse uterus treated with E2. Arrowhead indicates *Fam3d*. A published microarray dataset (GSE23072) was used in this analysis. The relative intensity of *Fam3d* was compared among the groups. Standard error of the mean (SEM) was calculated for each group (*n* = 3). The fold changes were determined by comparing the mRNA expression levels of each group to the levels of the wild-type vehicle group. The statistical significance was determined by one-way ANOVA followed by Tukey’s multiple comparison test (*** *p* < 0.001 versus vehicle). (**C**) RT-PCR and qRT-PCR analyses of *Fam3d* in the OVX mouse uterus at 24 h after treatment with vehicle, ICI, E2, and E2 + ICI. ICI treatment was administered 30 min before E2 treatment. Ribosomal protein L7 (*Rpl7*) was used as an internal control. SEM was calculated for each group (*n* = 3). The fold changes were determined by comparing the mRNA expression levels in each group to the levels of the vehicle group. The statistical significance was determined by one-way ANOVA followed by Tukey’s multiple comparison test (*** *p* < 0.001 versus vehicle). (**D**) Western blot analysis of FAM3D in the OVX mouse uterus at 24 h after treatment with vehicle, ICI, E2, and E2 + ICI. ICI treatment was administered 30 min prior to E2 treatment. β-actin was used as an internal control. Relative intensities were normalized to that of β-actin. SEM was calculated for each group (*n* = 3). The fold changes were determined by comparing the expression levels of each group to the levels of the vehicle group. The statistical significance was determined by one-way ANOVA followed by Tukey’s multiple comparison test (*** *p* < 0.001 versus vehicle). (**E**) Immunofluorescence staining of the OVX mouse uterus at 24 h after treatment with vehicle, ICI, E2, and E2 + ICI using FAM3D antibody (*n* = 3). Red represents FAM3D and blue represents DAPI. Scale bar, 20 μm. LE, luminal epithelium; GE, glandular epithelium; S, stroma; WT, wild type; ERαKO, estrogen receptor alpha knockout; veh, vehicle; n.s, not significant.

Estrogen plays a role in various signaling mechanisms by binding to ERα or ERβ, acting as a transcription factor or stimulating cellular signaling pathways. Alternatively, estrogen can activate intracellular signaling cascades through non-genomic actions via interactions with the G protein-coupled ER (GPER) [7]. Therefore, we investigated whether estrogen-mediated regulation of *Fam3d* expression in the mouse uterus is mediated by ER. To examine this, we compared the gene expression changes in the uteri of WT and ERαKO mice after E2 treatment using microarray data (GSE23072) [14]. The results showed that *Fam3d* expression increased 24 h after E2 treatment in normal WT mice, whereas there was no significant increase was observed in ERαKO mice (Figure 4A,B). This suggests that the E2-induced upregulation of *Fam3d* expression was mediated through ERα.

Next, we performed inhibition experiments using the ERα antagonist ICI. E2 was administered 24 h prior to sampling of OVX mice and ICI was administered 30 min before E2 treatment. The results showed that the E2-induced mRNA and protein expression of FAM3D were significantly reduced to basal levels by ICI treatment (Figure 4C,D). Immunofluorescence analysis also revealed that E2-induced FAM3D expression in the LE and GE was markedly inhibited by ICI treatment (Figure 4E). Taken together, these findings indicate that estrogen regulates FAM3D expression in the mouse uterus through an ERα-mediated pathway.

## 3. Discussion

Identification of uterine gene expression patterns that vary during the estrous cycle plays a crucial role in understanding female reproductive functions. Although the mouse estrous cycle and the human menstrual cycle are not identical, the core hormonal regulators and the associated tissue responses, including epithelial proliferation and differentiation, are highly conserved. The present study provides insights into the roles of female hormones, such as estrogen and progesterone, that influence the estrous cycle, as well as the mechanisms regulating uterine gene expression. As estrogen and progesterone levels are closely related to implantation and pregnancy maintenance [15,16], such studies can contribute to facilitating normal childbirth. Additionally, as these hormones are associated with various diseases, such as breast cancer, ovarian cancer, endometrial cancer, and endometriosis [17], these studies can shed light on their associations with these diseases and offer new approaches to prevention and treatment. Recent pan-cancer analyses link FAM3 genes to tumor progression and immune microenvironment remodeling, underscoring their broad clinical relevance [18,19]. Given these potential pathological roles, this field of research is important for enhancing our understanding of female reproduction and health, as well as for providing insights into managing related diseases.

Gene expression profile analysis of the mouse uterus during the estrous cycle identified four clusters with distinct expression patterns. Among these, we focused on proestrus, and estrus, the stages primarily regulated by estrogen. GO analysis revealed that the highly expressed genes in these two stages were commonly associated with immune processes. These results indicate that during proestrus and estrus, estrogen primarily regulates processes related to the immune system.

Changes in innate immunity based on the stages of the estrous cycle have been reported. Uterine secretions contain various interleukins, G-CSF, GM-CSF, TNF-α, CCL2, CXCL1, and other cytokines, which were found to be highly expressed during proestrus and estrus but not during diestrus [20]. These cytokines play crucial roles in leukocyte recruitment, vascular formation, proliferation, and differentiation [21]. These results indicate that the changes in innate immunity in the uterus primarily increase before ovulation to facilitate reproduction. In particular, the production of G-CSF, an essential cytokine that enhances uterine endometrial thickness before ovulation and plays a critical role in reproduction, was significantly high during the proestrus stage [22,23]. Thus, during early uterine proliferation, cytokines support the preparation of the uterine environment for reproduction and facilitate uterine receptivity, implantation, and fertility.

Fam3d was discovered by Zhu et al. in 2002 as a member of a cytokine-like gene family [9]. FAM3D functions as a chemotactic agonist for the G protein-coupled receptors FPR1 and FPR2, strongly attracting immune cells such as neutrophils and monocytes, thereby contributing to the pathogenesis of abdominal aortic aneurysms [12]. In the uterus, FPR1 is highly expressed in myometrial tissue and plays a crucial role in regulating myometrial contractions [24]. The present study found that the expression of FAM3D is induced by estrogen in endometrial epithelial cells. This suggested that FAM3D induces uterine contractions by interacting with FPR1 in the myometrium. Improper uterine contractions can lead to serious complications, including preterm labor. Therefore, understanding the mechanisms that regulate myometrial activity and identifying the related regulators could be highly beneficial for preventing preterm birth. Consistent with our findings, recent studies indicate that FAM3D regulates systemic glucose homeostasis, hepatic lipid deposition, and blood pressure by modulating endothelial nitric oxide synthase activity [25,26]. Further research is required to elucidate the exact role and mechanism of action of FAM3D in the uterus.

This study found that *Fam3b*, *Fam3c*, and *Fam3d* expression varied during the estrous cycle in the mouse uterus. *Fam3d* exhibited significantly higher mRNA expression during the proestrus and estrus stages but relatively lower expression during the metestrus and diestrus stages, with a similar pattern of protein expression. *Fam3b* and *Fam3c* also showed a gradual decrease in expression in the mouse uterus as the estrous cycle progressed. We plan to conduct further studies using a more detailed approach to investigate the protein levels of *Fam3b* and *Fam3c*, and their potential roles during different stages of the estrous cycle.

Considering the high estrogen secretion during the proestrus and estrus stages [5], we hypothesized that *Fam3d* expression could be regulated by estrogen. To test this hypothesis and exclude the influence of endogenous hormones, we conducted the experiments using OVX mice that were subsequently treated with E2. After 24 h of E2 treatment, *Fam3d* mRNA and protein expression were significantly induced in the OVX mouse uterus. Furthermore, the E2-induced *Fam3d* mRNA and FAM3D protein expression in the mouse uterus were blocked by the ERα antagonist ICI. This suggests that the mRNA and protein expression of *Fam3d* increased via the ER-mediated pathway. However, to study the mechanism in more detail, further experiments using selective ERα agonist PPT (1,3,5-tris (4-hydroxyphenyl)-4-propyl-1H-pyrazole) and selective ERβ agonist DPN (2,3-bis (4-hydroxyphenyl) propionitrile) will be necessary.

In addition, the proteins located in the nucleus and those present in the cytoplasm were different. These two compartments perform distinct functions within the cell, and the locations of proteins can greatly influence their roles and functions. Nuclear proteins are primarily involved in processes such as DNA replication and transcription, gene expression regulation, and RNA processing. They often regulate the expression of specific genes and are responsible for RNA synthesis and processing. In contrast, cytoplasmic proteins are mainly involved in cell signaling, metabolism, protein synthesis, and degradation. In the present study, FAM3D was exclusively expressed in the LE and GE of the mouse uterus and localized to the cytoplasm rather than the nucleus. Therefore, it is likely that FAM3D functions in cellular processes, such as signal transduction, rather than in regulation of gene expression within the cell.

Liang et al. reported FAM3D expression in the apical region of the crypt epithelial cells in the colon [11], which closely resembles the expression pattern observed in the mouse uterus. In the colon, FAM3D plays a role in homeostasis, protection against inflammation-related cancers, and maintenance of normal gut microbiota composition. Both the uterus and colon are lined with epithelium, which functions in protection, absorption, and secretion; moreover, they share common morphological characteristics, including the presence of mucus. Furthermore, in both the uterus and colon, epithelial cells induce the secretion of growth factors and cytokines [27,28]. Considering these similarities, it is plausible that FAM3D performs similar functions in the uterus and colon. However, further studies are required to elucidate the tissue-specific molecular mechanisms and functions of FAM3D.

In conclusion, the expression of FAM3D is dynamically regulated in the mouse uterus during the estrous cycle, and this regulation is mediated through the estrogen–ER signaling pathway. This study sheds light on a novel regulatory mechanism in the uterus and contributes to our understanding of the role of FAM3D. Further research may help elucidate the specific functions and implications of FAM3D in uterine physiology and reproductive processes.

## 4. Materials and Methods

### 4.1. Animals

ICR female mice (6–8 weeks old) were procured from JA BIO (Suwon, Republic of Korea). The mice were housed under controlled temperature and lighting conditions with a 12 h light/dark cycle and provided ad libitum access to food and water. Animal welfare and handling were conducted in compliance with the guidelines stipulated for the care and use of laboratory animals. The study protocol was approved by the Institutional Animal Care and Use Committee (IACUC, Approval No. KU22074).

### 4.2. Estrous Cycle Stage Determination

A vaginal smear assay was performed to determine the estrous cycle in the mice. DPBS was injected into the vagina and the fluid was collected by pipetting several times. The collected fluid was spread onto a slide and dried on a heat block. The slides were stained using a Diff-Quick staining kit (Sysmex, Kobe, Japan). The estrous cycle was determined by analyzing the type and number of cells on slides under a ZEISS Primo Star microscope (ZEISS, Oberkochen, Germany) at 10× magnification. Once the estrous cycle was identified, 3 mice were sacrificed at each stage and the uterus was excised for further analysis.

### 4.3. Estrogen Treatments

To study the impact of ovarian steroid hormones on gene and protein expression, six-week-old adult female mice were subjected to ovariectomy and allowed to rest for 2 weeks to deplete endogenous hormones. Thereafter, either vehicle (0.1 mL sesame oil; Acros Organic, Geel, Belgium) or β-estradiol (E2; 300 ng/mouse; Sigma-Aldrich, St. Louis, MO, USA) in sesame oil was administered via a single subcutaneous injection, and the mice were sacrificed at varying time intervals (2–24 h) post-injection (*n* = 3). To investigate the involvement of nuclear ER signaling, ovariectomized (OVX) mice were pretreated with a single subcutaneous injection of the ER antagonist ICI 182,780 (500 μg/mouse; MedChemExpress, Monmouth Junction, NJ, USA) in sesame oil, 30 min before E2 treatment. This experimental design enabled evaluation of the time-dependent effects of E2 and the role of nuclear ER signaling in regulating gene and protein expression in the mouse uterus.

### 4.4. RNA Extraction, RT-PCR, and qRT-PCR

To obtain high-quality RNA from mouse uterus and various tissues, we used a method involving rapid freezing of freshly collected uteri in liquid nitrogen followed by individual homogenization of each tissue in RLT buffer (Qiagen, Hilden, Germany) containing a stainless steel bead using the Tissue Lyser LT homogenizer (Qiagen). The homogenized samples were then centrifuged at 13,000 rpm for 3 min to remove debris before proceeding with the RNeasy mini kit (Qiagen) protocol, which involves column-based RNA purification. DNase I (NEB, Ipswich, MA, USA) treatment was performed to eliminate any potential genomic DNA contamination, and the RNA was eluted in 30 μL of RNase-free water. RNA concentration and purity were determined using a NanoDrop 2000 spectrophotometer (Thermo Fisher Scientific, Waltham, MA, USA). The obtained RNA was then reverse-transcribed into cDNA using the SensiFAST TM cDNA Synthesis Kit (Meridian Bioscience, Newtown, OH, USA) according to the manufacturer’s instructions, and the resulting cDNA was diluted with RNase-free water and used as a template for PCR and qPCR analysis. Quantitative real-time RT-PCR (qRT-PCR) was performed using the PowerUp SYBR Green Master Mix (Thermo Fisher Scientific) on a QuantStudio 1 Real-Time PCR Instrument (Applied Biosystems, Waltham, MA, USA). We used *Rpl7*, a ribosomal protein L7, as the reference gene for normalization of the target gene Ct values. The following primer sequences were used: *Fam3b* (NM_020622.3; 152bp): GCAGGTAGGCCAGCTTTCAT (forward), and TACCCAGAATACCAGGCGCA (reverse); *Fam3c* (NM_138587.4; 138bp): ACGGTCTTGCTCATGCACAC (forward), and CCAGAAGCAAGACTGACGGG (reverse); *Fam3d* (NM_146050.2; 131bp): CGGACTTGAGAGCAGAACCC (forward), and TGCTTCCTTAGACGGGAAGTG (reverse); *Rpl7* (NM_011291.5; 122bp): GTCTTCCCTGTTGCCAGCAT (forward), and GGAAACGCTTCAAGGAAGCAA (reverse).

### 4.5. Western Blot Analysis

To obtain protein extracts from mouse uterus, individual uterine tissues were collected and immediately frozen in liquid nitrogen. Afterwards, the tissues were separately homogenized using a Tissue Lyser LT homogenizer (Qiagen, Hilden, Germany) with 200 μL of RIPA buffer containing a mixture of protease inhibitor and phosphatase inhibitor (NaF 50 mM, Na_3_VO_4_ 5 mM, β-glycerophosphate 50 mM) and a stainless steel bead to facilitate tissue disruption. Homogenized samples were incubated on ice for 30 min, with vortexing every 5 min to further aid in protein extraction. Following centrifugation at 13,000 rpm for 20 min at 4 °C, the supernatant containing the protein extracts was carefully transferred to a new tube. Next, aliquots of the protein extracts were loaded onto an SDS–polyacrylamide gel (10–12%) and electrophoresed to separate the proteins based on their molecular weights. The separated proteins were then transferred to a PVDF membrane using the wet tank method. The membrane was blocked with 5% skim milk (BD Biosciences, Milpitas, CA, USA) for 2 h at room temperature, followed by an overnight incubation at 4 °C with FAM3D goat polyclonal antibody (1:1000, AF3027, R&D Systems, Minneapolis, MN, USA) and beta Actin mouse monoclonal antibody (C4) conjugated to HRP (1:10,000, sc-47778 HRP, Santa Cruz, CA, USA) to detect the proteins of interest. After washing the membrane with a 0.1% TBS-T, it was incubated with Rabbit anti-Goat IgG (H + L) Secondary Antibody and HRP (1:10,000, 61-1620, Invitrogen, Carlsbad, CA, USA) for 1 h at room temperature. Detection of the proteins of interest was achieved using SuperSignalTM West Femto Maximum Sensitivity Substrate (34095, Thermo Fisher Scientific, Waltham, MA, USA) and West Pico PLUS Chemiluminescent Substrate (34580, Thermo Fisher Scientific, Waltham, MA, USA), which emitted chemiluminescence that was visualized using the ChemiDOCTM XRS+ system (Bio-Rad, Hercules, CA, USA). To quantify the expression levels of the proteins of interest, the density of the blotting bands of FAM3D was measured and normalized using Total Beta-actin bands as the internal control. The analysis was performed using the ImageJ Fiji platform (version 2.11.0).

### 4.6. Immunofluorescence Staining

To investigate the cellular distribution and localization of FAM3D after E2 treatment, the mouse uteri were fixed in 4% paraformaldehyde and embedded in Paraplast X-TRA (Sigma-Aldrich). Thin uterine sections (5 μm) were then deparaffinized, rehydrated, and subjected to antigen retrieval in 10 mM sodium citrate buffer at pH 6.0 for 20 min. To block nonspecific binding, the sections were incubated in 5% BSA in Tris-buffered saline with 0.3% TBS-T for 1 h. Next, the sections were incubated overnight at 4 °C with FAM3D goat polyclonal antibody (1:50; AF3027, R&D Systems). After washing with 0.1% TBS-T, sections were incubated with Donkey anti-Goat IgG (H + L) Cross-Adsorbed Secondary Antibody and Alexa Fluor 555 (1:1000; A-21432, Invitrogen) for 1 h at 25 °C. Finally, the sections were washed with 0.1% TBS-T and mounted on slides with Mounting Medium with DAPI–Aqueous Fluoroshield solution (Abcam, Cambridge, UK) for observation.

### 4.7. Whole-Genome RNA Sequencing (RNA-Seq)

For RNA-seq analyses, two biological replicates were sequenced for each stage of the estrous cycle. Poly(A)+ mRNA was purified from 1 μg of total RNA using oligo(dT) magnetic beads and used to generate TruSeq Stranded mRNA libraries (Illumina, San Diego, CA, USA). Briefly, purified mRNA was fragmented, reverse-transcribed to first-strand cDNA using random hexamers, and converted to double-stranded cDNA, followed by end repair, A-tailing, adapter ligation, and PCR amplification. Libraries were quality-checked on an Agilent 2100 BioAnalyzer (Agilent, Santa Clara, CA, USA), quantified with the KAPA Library Quantification Kit (Kapa Biosystems, Wilmington, MA, USA), and sequenced as 151-bp paired-end reads on an Illumina NovaSeq X Plus (Illumina, San Diego, CA, USA). RNA-seq datasets were aligned to the Ensembl transcript database (mm10) using the STAR tool (v2.7.11a). The mapped reads were counted using featureCounts, a read count summarization function in the R subread package (version 2.16.1). Differential gene expression analysis was performed using DESeq2 in R/Bioconductor with the following parameters: base mean count > 15 and false discovery rate (FDR) < 0.05. Principal component (PCA) analysis was performed using ggplot2 (DESeq2 package; version 1.42.1) for clustering the samples based on gene expression patterns. Heat maps were generated using the Complex Heatmap package in R (version 2.18.0). Functional annotation analysis with differentially expressed genes (DEGs) was performed using DAVID (v6.8).

### 4.8. Reassessment of Public Datasets

Public microarray dataset obtained from mouse uteri of wild-type (WT) and ERα knockout (ERαKO) mice after E2 treatment (GSE23072) was downloaded and analyzed using the interactive and publicly available web tool GEO2R (https://www.ncbi.nlm.nih.gov/geo/info/geo2r.html, accessed on 15 September 2022). In the present study, genes that were differentially expressed between WT and ErαKO mice were screened using an adjusted value of less than 0.05 and a |log fold change| of >1.0 as thresholds.

### 4.9. Statistical Analysis

The results are reported as mean value ± standard error of the mean (SEM). Statistical analyses were conducted using one-way analysis of variance (ANOVA), followed by Tukey’s post hoc test for multiple comparisons. Analyses were performed using the online tool One-Way ANOVA Test calculator from the Statistics Kingdom [29].

### 4.10. Declaration of AI

No generative AI was used for data, images, or discussions; all content was reviewed and verified by the authors.

## Data Availability

The RNA-seq data used in this study are a fundamental component of our research and are available in [GSE241420].

## Supplementary Materials

The following supporting information can be downloaded at: https://www.mdpi.com/article/10.3390/ijms262411840/s1.

## Abbreviations

The following abbreviations are used in this manuscript:

DEGs	Differentially expressed genes
E_2_	17β estradiol
ER	Estrogen receptor
FDR	False discovery rate
GE	Glandular epithelium
GPER	G protein-coupled estrogen receptor
LE	Luminal epithelium
OVX	Ovariectomized
