# Supplementary material for: Estrogen-Mediated Regulation of Fam3d in Mouse Uterus During the Estrous Cycle
Source: Int J Mol Sci. 2025 Dec 8;26(24):11840. doi: 10.3390/ijms262411840 (PMC12732396; doi:10.3390/ijms262411840)
Supplement: Supplementary file 1 [file ijms-26-11840-s001.zip › ijms-3998552-supplementary.pdf]

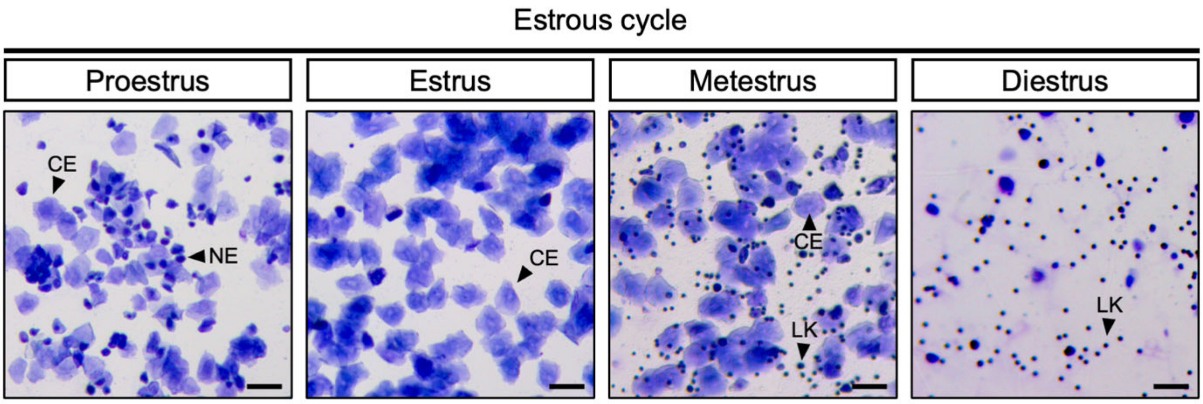

Supplementary Figure S1. Representative images for the estrous cycle stages, determined by vaginal smear assay. Scale bar, 50 µm. NE, nucleated epithelial cell; CE, cornified epithelial cell; LK, leukocyte.

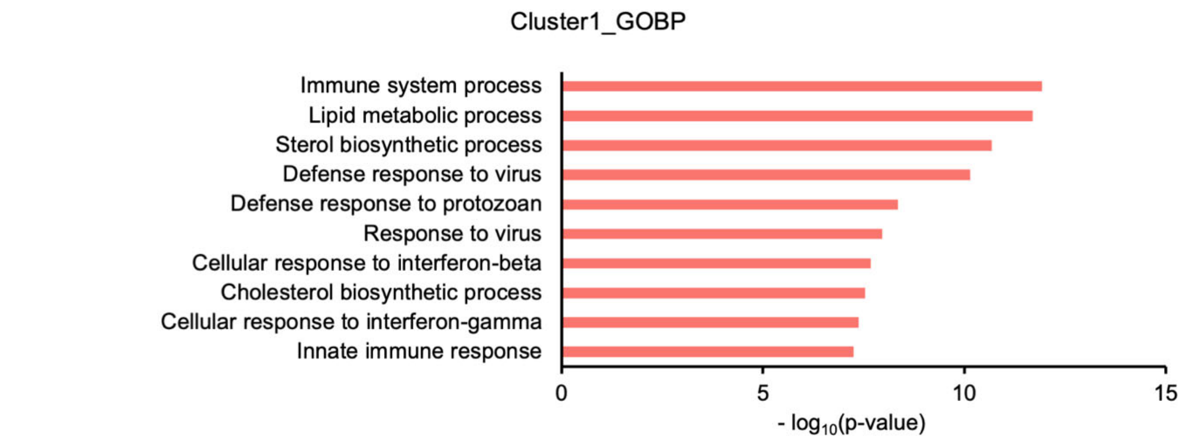
Cluster1_GOBP
Immune system process
Lipid metabolic process
Sterol biosynthetic process
Defense response to virus
Defense response to protozoan
Response to virus
Cellular response to interferon-beta
Cholesterol biosynthetic process
Cellular response to interferon-gamma
Innate immune response
0
5
10
15
- $\log_{10}$(p-value)

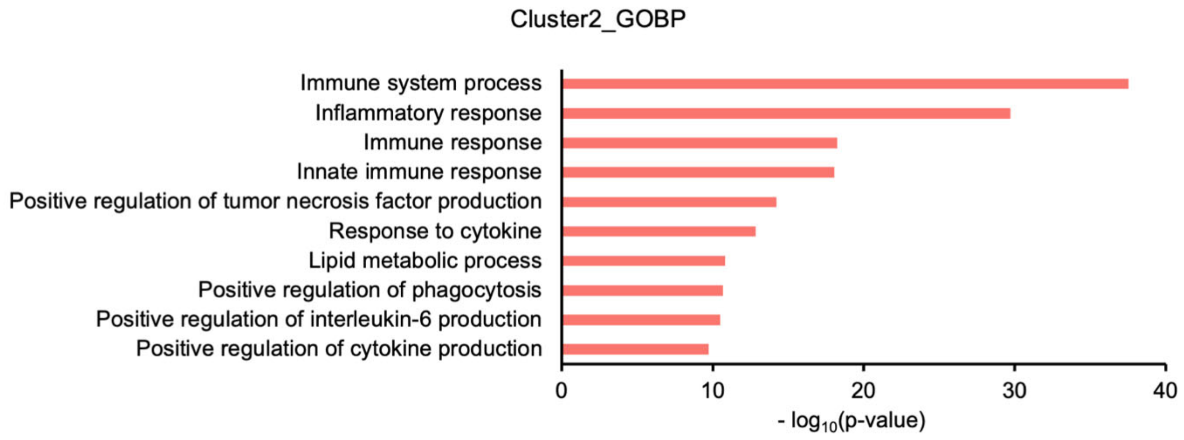
Cluster2_GOBP
Immune system process
Inflammatory response
Immune response
Innate immune response
Positive regulation of tumor necrosis factor production
Response to cytokine
Lipid metabolic process
Positive regulation of phagocytosis
Positive regulation of interleukin-6 production
Positive regulation of cytokine production
0
10
20
30
40
- $\log_{10}$(p-value)

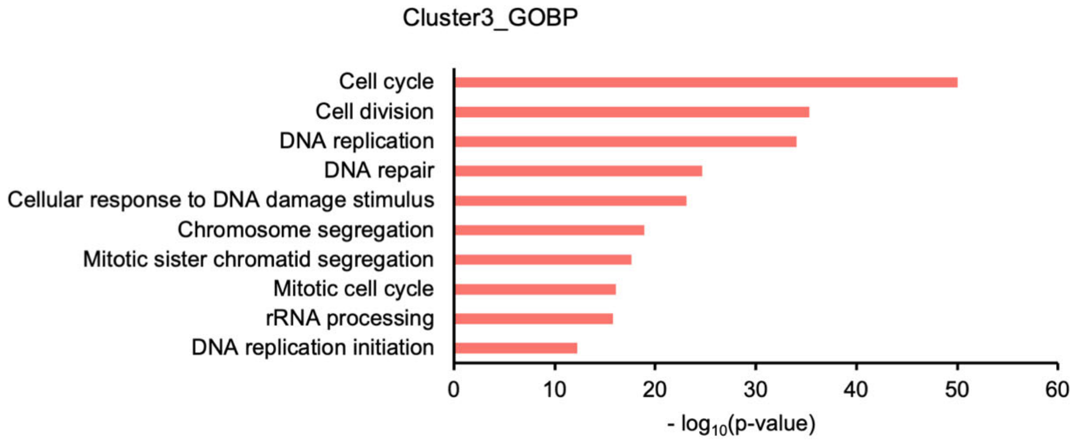
Cluster3_GOBP
Cell cycle
Cell division
DNA replication
DNA repair
Cellular response to DNA damage stimulus
Chromosome segregation
Mitotic sister chromatid segregation
Mitotic cell cycle
rRNA processing
DNA replication initiation
0
10
20
30
40
50
60
- $\log_{10}$(p-value)

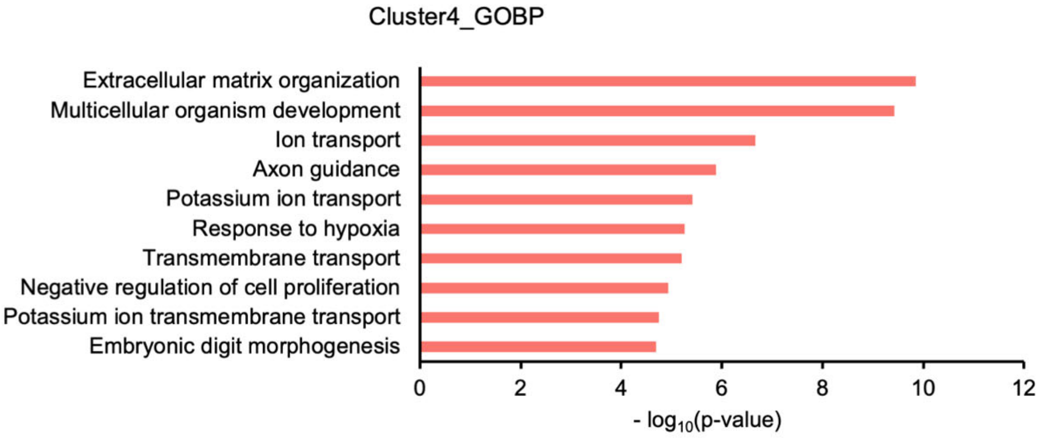

Supplementary Figure S2. Gene Ontology Biological Process (GOBP) analysis was conducted for each cluster with differently expressed genes in the mouse uterus during the estrous cycle. The y-axis represents significantly enriched biological process (BP) categories in GO analysis relative to the target genes, and the x-axis shows the enrichment scores of these terms.
